# Ferrocene Introduced into 5-Methylresorcinol-Based Organic Aerogels

**DOI:** 10.3390/polym12071582

**Published:** 2020-07-16

**Authors:** Ludmila V. Erkhova, Igor A. Presniakov, Michail I. Afanasov, Dmitry A. Lemenovskiy, Haojie Yu, Li Wang, Mati Danilson, Mihkel Koel

**Affiliations:** 1 Department of Chemistry, Moscow State University, Lenin Hills, 1\3, 119991 Moscow, Russia; ludmilka95@rambler.ru (L.V.E.); Ipresniakov1969@mail.ru (I.A.P.); pf@radio.chem.msu.ru (M.I.A.); dali@org.chem.msu.ru (D.A.L.); 2 State Key Laboratory of Chemical Engineering, College of Chemical and Biological Engineering, Zhejiang University, Hangzhou 310027, China; hjyu@zju.edu.cn (H.Y.); wanglili@rdgy.cn (L.W.); 3 Department of Material and Environmental Technology, School of Engineering, Tallinn University of Technology, Ehitajate 5, 19086 Tallinn, Estonia; mati.danilson@taltech.ee; 4 Department of Chemistry and Biotechnology, School of Science; Tallinn University of Technology, Ehitajate 5; 19086 Tallinn, Estonia

**Keywords:** organic aerogel, resorcinol–formaldehyde aerogel, ferrocene, supercritical drying

## Abstract

The polycondensation sol–gel reaction of 5-methylresocinol and formaldehyde with additional compounds in reaction media is a relatively simple way to produce modified aerogels. In order to obtain aerogels with a large surface area and high porosity, the conditions for gel formation, the solvent exchange process before drying, and the supercritical drying process were optimized. A successful attempt was made to introduce ferrocene units into 5-methylresocinol-formaldehyde-based aerogels. The resulting aerogels are amorphous substrates, and no aggregated ferrocene units were found in their structures. All of the aerogel samples that were obtained are structurally similar despite differences in the original ferrocene units and their initial concentration. It was found that the inclusion limit of ferrocene structural blocks into an aerogel is ~6% wt. The structures of the inclusions in which all of the Fe atoms in the aerogel substrates were present in ferrocene/ferrocenium at an approximate ratio of 60/40 to 55/45 were confirmed by X-ray photoelectron spectroscopy and Mössbauer spectroscopy. Aerogels with ferrocene/ferrocenium inclusions are likely to exhibit reversible redox activity in reactions with gaseous reagents.

## 1. Introduction

Aerogels—the nanoscale skeletal structure of which forms a solid macroscopic state—are known to exhibit unique properties. It has been proposed that highly nanoporous aerogels be recognized “as a state of matter rather than as a functional material, because of its qualitative differences in bulk properties, transitional density and enthalpy between liquid and gas and diverse chemical compositions” [1].

Resorcinol–formaldehyde (RF) aerogels are well-known organic aerogels with various applications, mainly as precursors to electrically conductive carbon aerogels [2,3,4]. RF aerogels are typically prepared using Pekala’s method, followed by drying with supercritical CO_2_ [5]. However, an acid-catalyzed gelation process allows the reaction to be completed in minutes rather than days [6] and by modifying the classical sol–gel synthesis route it is possible to obtain flexible aerogels [7].

Supercritical drying preserves the original porosity of the hydrogel to a substantial degree and can be used on an industrial scale [8]. Thus, supercritical drying is often the preferred method that drives the development of drying process parameters [9,10]. The aerogel preparation method using supercritical drying usually includes solvent exchange before drying, with proper process parameters in order to avoid a non-repeatable process and wide variation in yields [11].

The characteristic properties of aerogels are high porosity (more than 90%), a large surface area (up to 1000 m^2^ g^−1^), low envelope density (0.03–0.5 g/cm^3^) and low thermal conductivity (as low as 0.012 W/m K) [12]. However, different aerogels have very different characteristics.

One of the strategies for incorporating metal species into the structure of an aerogel involves a resorcinol derivative that can be copolymerized using sol–gel techniques. One example of doping metals is the use of reagents containing an ion exchange moiety that permits the required metal to be attached subsequently [13]. This enables the creation of a novel functional material platform as a compound bearing the metal center in the main chain or side chain of the polymer architecture. Various dopants with metal content that maintain the basic characteristics of an unmodified aerogel can open a wide variety of possibilities for advanced catalysts and allow for catalytic selectivity to promote one reaction over another [14,15,16,17]. In recent years, metal–organic frameworks (MOFs) as a means of introducing metals into aerogel structures have attracted great interest, and MOF-based hydrogels and aerogels have shown results that outperform common MOF materials in many respects [18].

Of the various organometallic materials, ferrocene and its derivatives have received much attention due to their electrochemical activity, liquid crystallinity and thermal and photochemical stability [19,20]. Ferrocene is capable of switching between two oxidation states in a reversible manner, with both the reduced and oxidized forms being chemically stable [21]; thus, the incorporation of ferrocene into the polymeric system introduces different redox, optoelectronic and magnetic properties [22,23]. Some of the new materials are good candidates for use as storage materials due to their high redox reversibility and excellent charge/discharge performance [24,25]. It is thus reasonable to expect that these materials and our aerogels among them could be used as redox-responsive storage devices and indicative sensors.

The incorporation of ferrocene into side chains along a polymeric backbone may be achieved in two ways: either the polymer can be constructed from monomeric units that already bear the metallocenes, or the aerogel can be functionalized at a later stage. However, this process is complicated due to the difficulty of reaching the surface of the entire substrate and therefore usually requires pretreatment of the surface. The only known example involved the absorption of ferrocene into the RF gel during preparation, and the linkage of ferrocene with the aerogel substrate was not proved [26].

The aim of this study was to introduce into the structure of aerogels chemically bonded ferrocene moieties using common RF aerogel preparation technology. The object was to obtain 5-methylresorcinol–formaldehyde aerogels containing ferrocene without making substantial changes to the common preparation methodology. In the present work, two monomeric precursors that contained ferrocene and can contribute to gel formation were used—ferrocenyl methanol (FcCH_2_OH) and ferrocenyl methyl phenyl ether (FcCH_2_OPh). An acidic catalyst was used to aid gel formation. Both compounds exhibit characteristics of electrophile reagents, especially in slightly acidic media. Under these reaction conditions it was expected that the ferrocene moieties would act as phenolic components, as well as cross-linking units resembling formaldehyde, thus enabling the incorporation of at least 10 mol% of the ferrocene units into the structure of the polymer. The ultimate goal of this study was to be able to include controlled amounts of ferrocene units into resorcinol–formaldehyde polymer using ordinary procedures for obtaining RF aerogels in order to produce electrochemically active material. To the best knowledge of the authors this is the first instance of preparing aerogels with ferrocene units chemically bonded to the aerogel structure.

## 2. Materials and Methods

Two units containing ferrocene—ferrocenyl methanol (FcCH_2_OH) and ferrocenyl methyl phenyl ether (FcCH_2_OPh)—were used in this project.

Ferrocenyl methanol with 97% purity was obtained from Sigma-Aldrich (Darmstadt, Germany). Ferrocenyl methyl phenyl ether was prepared in the laboratory (see Supplementary material).

For the preparation of gels, 5-methylresorcinol (MR) with >99% reported purity was provided by AS VKG (Estonia); HCl 38% super-pure was obtained from Etec, Russia. A Milli-Q water system was used to purify the water. Formaldehyde (37 *w/w* solution in water) was purchased from Sigma-Aldrich (Darmstadt, Germany).

Two types of gels were prepared: Gel 1 with ferrocenyl methyl phenyl ether (Table S1 in Supplementary Materials) and Gel 2 with ferrocenyl methanol (Table S2). The protocols were based on common RF preparation recipes and were very similar—units containing ferrocene were dissolved in ethanol; 1-M hydrochloric acid was used as a catalyst; gelation was carried out at 65–68 °C. The sole difference pertained to the use of 5-methylresorcinol instead of resorcinol. After the gel was formed, it was aged one week before further processing; the solvent exchange with acetone was performed before supercritical drying.

Supercritical CO_2_ extraction was performed on a 70-mL thermostated high-pressure reactor from LIK Co., Ltd. (Moscow, Russia), and a TharSFC high-pressure pump was used.

An optimized drying regime was used for drying the resulting gels (see Supplementary material).

Structural and thermal characterization of the polymers was carried out by means of a Fourier transform infrared spectroscopic (FTIR) analysis using a Tensor 27 (Bruker, Karlsruhe, Germany) FT-IR spectrometer in the 400 to 4000 cm^−1^ range by the KBr disk method and a thermogravimetric analysis (TGA) was conducted using a Netzsh simultaneous thermal analyzer (TG-DSC apparatus) in an N_2_ atmosphere with a heating rate of 10 K/min from 25 °C to 900 °C.

The Fe content of the material was measured by a Spectra AA 220F flame atomic absorption spectrometer (AAS) (Varian, Palo Alto, CA, USA).

The nitrogen adsorption analyses were performed using a sorptometer KELVIN 1042 (Costech International) with helium used as the carrier gas and nitrogen as the adsorptive gas. Nitrogen adsorption data were collected at relative pressures from 0 to 1 and at liquid nitrogen temperature (the mode of analysis was “fast”). The specific surface areas (S_bet_) and (S_L_) were calculated according to the Brunauer–Emmett–Teller theory and measured Langmuir isotherm, respectively. The specific micropore (V_mic_) and total pore volumes (V_tot_) were determined by a *t-*plot. The uncertainty of the method is ≤3%.

A Zeiss FEG-SEM Ultra 55 microscope was used (with accelerating voltages between 5 and 30 kV) for scanning electron microscopy (SEM) and energy-dispersive X-ray spectroscopy (EDS). (The SEM samples were coated with gold for 100 s at 30 mA using a sputter coater).

The X-ray diffraction (XRD) pattern was recorded with a Rigaku Ultima IV diffractometer using a D/teXUltra line detector (Woodlands, TX, USA). Cu-Kα radiation was used with a Ni filter for removing Kβ radiation. The recorded diffractograms were analyzed by Rigaku PDXL software.

The X-ray photoelectron spectroscopy (XPS) spectra were obtained with the Kratos Analytical, Ltd. (Manchester, UK). X-ray photoelectron spectroscopy system Axis Ultra DLD equipped with a monochromatic Al Kα X-ray source and an achromatic Mg Kα/Al Kα dual anode X-ray source. The monochromatic Al Kα anode (1486.6 eV) was operated at 150 W and 15 kV. Low-energy electrons were used for charge compensation to neutralize the sample. The 180° hemispherical energy analyzer with an average radius of 165 mm was operated using a hybrid lens mode at a pass energy of 160 eV for survey spectra and 20 eV for regions spectra. The binding energy values were calculated on the basis of the C 1 s peak at 284.6 eV. The relative atomic concentrations of the elements were determined from the appropriate integrated peak areas at the core level and the sensitivity factors provided by the original analysis Kratos Vision 2.2.10 software. Shirley background subtraction was used to calculate the relative atomic concentrations.

Mössbauer spectroscopic measurements were carried out using an electrodynamic spectrometer MS-1104m (Southern Federal University, Rostov-on-Don, Russia), operating in constant acceleration mode with a 1.1 GBq *γ*-source of ^57^Co(Rh) kept at room temperature while the studied samples were introduced into a liquid nitrogen cryostat, which was used to vary the temperature between 78 К and 310 К. The spectra were fitted using the SpectrRelax program [27]. The isomer shift values were calculated relative to that of a-Fe at 300 K.

## 3. Results

### 3.1. IR Spectra

The IR spectra are not very informative at the present stage. A comparison of the IR spectra of different aerogels reveals a close similarity of Fc-aerogels to the resorcinol–formaldehyde aerogels studied previously (Figure S1 in Supplementary Materials).

The wide peak at 3438 cm^−1^ of the stretching −OH groups may be attributed to the water encapsulated in the sample (Figure S2). The main characteristic peaks of ferrocene in the range of 2000 to 1000 cm^−1^ overlapped with the main peaks from the polymer matrix (Figure S3). The stretching vibration of C–H in the ferrocene and phenyl rings was observed at 3052 cm^−1^; signals around 490, 1490 and 1140 cm^−1^ were assigned to the Cp-Fe stretching mode, C–C of cyclopentadienyl bending and breathing vibration, respectively [28].

### 3.2. Fe content in Aerogel

At the outset it was important to estimate the Fe content of the aerogel and subsequently to use spectroscopic methods to determine the configuration of the Fe units in the polymer matrix. The results of measurements of Fe content by different methods are presented in Table 1.

The aerogel has an homogeneous structure, which makes it reasonable to assume that surface measurements like EDS and XPS could be interpolated to whole polymer matrices. The S/N relationship for Fe XPS peaks is more than 10, which makes these measurements dependable despite a slightly higher level of uncertainty.

The electrochemical activity of the material is dependent on active groups in the polymer. This makes it critical to control the number of active groups in the ferrocene units. The data presented in Table 1 confirms that the Fe content can be controlled by the amount of Fc units added to the reaction mixture. In separate trials it was found that under the present reaction conditions incorporating more than 7.5 mol% of the ferrocene units into the structure of the polymer was not possible. The iron atoms in the referenced aerogels clearly exist in the form of sandwich complexes: the presence of ferrocene and ferrocenium was validated by spectroscopic studies.

One of the evident reasons for these weight limits is of a methodological nature. Under the usual conditions of 5-methylresorcinol–formaldehyde gel synthesis the amount of ferrocene units is limited by their low solubility. Therefore, work is being undertaken to develop a new protocol for mixed copolymer synthesis.

### 3.3. TGA Results

The thermal behavior of the aerogels was tested by TGA in an inert atmosphere. The results show a general course of degradation characteristic of resorcinol–formaldehyde aerogels [29]—a similar smooth process of degradation for every sample (Figure S4)).

MR-FA aerogels are a well-known source of carbon aerogel; subject to further studies, we hypothesize that given a weight loss of around 45% the residue could constitute a porous carbon aerogel with Fe nanoparticles [30].

### 3.4. Nitrogen Adsorption Analysis and Porosity

At this stage of the study it was important to confirm that the resulting aerogel possessed the characteristic porosity and surface area. The resulting materials had low density and high porosity. A porosity analysis by nitrogen adsorption led to the conclusion that all the aerogel samples could be characterized as mesoporous and they did not indicate the presence of microporosity (Table 2). However, the surface area was lower than that of regular MR-FA aerogels, in which the S_BET_ is ~450 m^2^/g and the V_tot_ is ~900 mm^3^/g [31].

### 3.5. SEM and EDS Results

**Figure 1:**
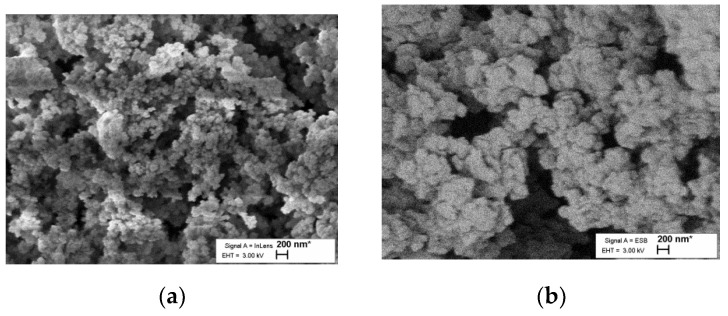

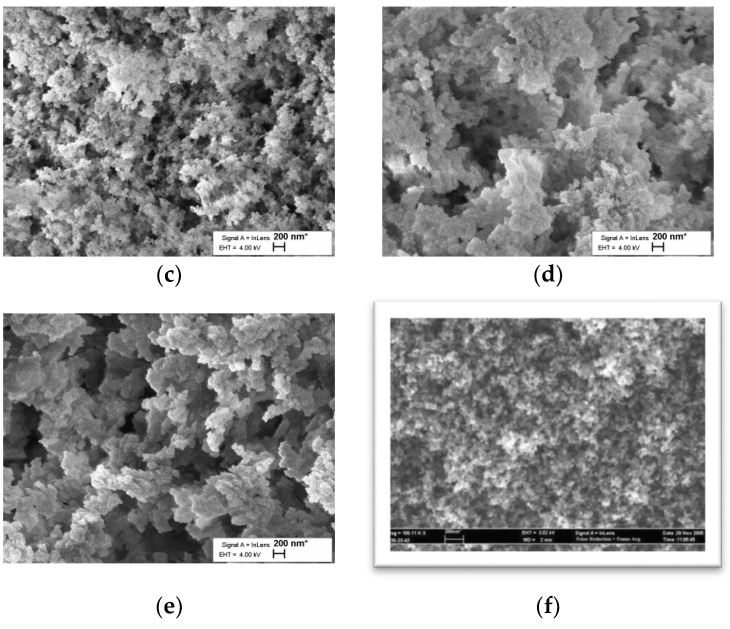
SEM images of (**a**) samples 1a, (**b**) sample 3a, (**c**) sample 1b, (**d**) sample 2b and (**e**) sample 3b with identical magnification. (**f**) sample 4 is a typical RF aerogel prepared under similar conditions and in the same scale.

The SEM image in Figure 1 shows that the surface morphology of the material is similar to a typical resorcinol–formaldehyde aerogel [31].

The EDS spectra are similar for every sample examined (Figure S5); they clearly show the existence of Fe, confirming the presence of iron in the structure of the material.

The X-ray diffraction (XRD) analysis confirmed that the material is amorphous, with no sharp peaks in the diffractogram (Figure S6).

### 3.6. XPS Results

The chemical composition and oxidation state of the surface of the Fc-aerogel samples were investigated by X-ray photoelectron spectroscopy (XPS) in order to identify the ferrocene units incorporated in the aerogel structure. The XPS survey spectrum of Sample 3b is shown in Figure S7 as an example. The main elements detected on the surface of all the studied Fc-aerogel samples were O, C and Fe. As HCl was used as a catalyst in the synthesis of the aerogels, residues of Cl are also seen in the spectra. The positions of the photoelectron peaks, atomic concentrations and assignments are shown in Table S5.

Deconvolution of O1 s spectra (Table S3) indicated that the surface oxygen was distributed as C=O (oxygen atom of a carbonyl group), C–O (linked oxygen atom) and O–C=O (oxygen atom of a carbonyl group + linked oxygen atom) groups. The percentage of the C–O group in the applied organic aerogels was higher than that of other surface groups containing oxygen. In general, the groups containing oxygen are known to impart organic aerogel catalytic sites [16].

**Figure 2:**
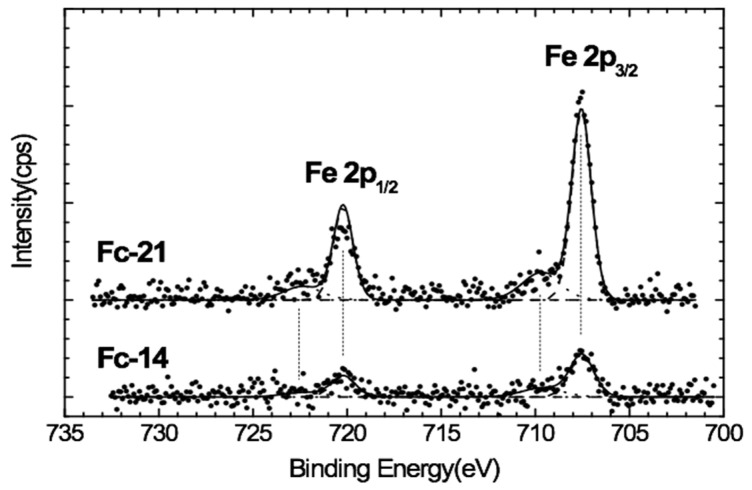
Peaks related to Fe 2p on the XPS spectra of Samples 1a (Fc-14) and 3b (Fc-21).

The narrow region spectra for Fe 2p of the 3b and 1a samples are shown in Figure 2. A positive offset is applied to the 3b spectrum, but the ratio of intensity to the 1a spectrum was kept comparable. The spectra show prominent sharp features at 707.6eV and 720.4eV accompanied by broad shoulders at higher energies, which are associated with Fe 2p3/2 and 2p1/2, respectively. Experimental data show the spin orbit splitting only at 12.8eV. The iron comprises two oxidation states: Fe^2+^ and Fe^3+^. The lowest binding energy peak at 707.6eV is attributed to the Fe^2+^ of the ferrocene unit grafted onto the aerogel surface, which agrees with the literature on compounds that have ferrocene in their structure [32]. Since Fe^2+^ is a low-spin configuration, no satellite feature is observed. The broad structure at a higher binding energy (about 709.7eV) of the main Fe 2p3/2 peak is not a satellite structure but is rather due to Fe^3+^ states from oxidation of the surface exposed to air. It could also be related to the presence of ferrocenium, which is typical of these systems, due to manipulation of the samples or to irradiation by X-ray beam [33]. These results indicate that most of the ferrocene Fe ions in the material were in an oxidation state of +2 (due to the binding energy of 709.7) and the 722.7 eV peak is attributed to the Fe^3+^ state of the iron [34].

### 3.7. Mössbauer Measurements

In our study, Mössbauer measurements were not able to detect in the aerogel structure any other forms of Fe than those that were present in the ferrocene complex (two cyclopentadienyl rings bound on opposite sides of a central iron atom). The Mössbauer spectra of the substituted ferrocenes Fc1—(C_5_H_5_)Fe(C_5_H_4_CH_2_OH) and Fc2—(C_5_H_5_)Fe(C_5_H_4_CH_2_O-Ph) are well-resolved doublets at all temperatures in the range of 78–310 K. A typical spectrum at 78 K is shown in Figure S8; the hyperfine parameters are included in Table S4. It is generally accepted [35] that in ferrocene (C_5_H_4_R)Fe(C_5_H_5_) derivatives, the substituents R have little effect on both the isomeric shift (δ) and the quadrupole splitting (Δ). The spectral parameters of compounds Fc1 and Fc2 are typical of substituted ferrocene [36]. It should be noted that the isomeric shift and quadrupole splitting of the Fc1 and Fc2 spectra are close to the parameters of the doublet component of the spectra of polymers.

**Figure 3:**
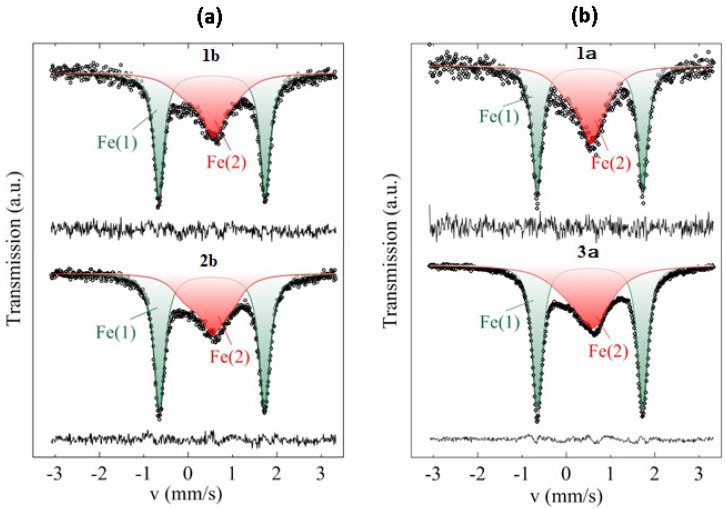
Experimental 57Fe Mössbauer spectra (hollow dots) of Samples 1b and 2b (**a**) and 1a and 3a (**b**) recorded at 78 K. The solid lines are simulations of the experimental spectra as superpositions of the Fe(1) and Fe(2) subspectra as described in the text.

The ^57^Fe Mössbauer spectra of Samples 1b, 2b, 1a and 3a measured at 78 K (Figure 3) can be described as a superposition of two partial subspectra Fe(i) with different hyperfine parameters, as shown in Table 3. The isomer shift (δ_1_) and quadrupole splitting (Δ_1_) of the first subspectrum Fe(1), which are represented by a well-resolved quadrupole doublet, correspond to the low-spin ferrous cations Fe^2+^(*d*^6^) in ferrocene-related compounds [37].

The broad non-Lorentzian shape of the Fe(2) subspectrum is characteristic of relaxation behavior. This could be related to the paramagnetic iron ions with an effective spin of *S*_z_^eff^ = ±5/2 fluctuating up and down along the major symmetry axis when ferrocene-related systems undergo one-electron oxidation Fe^2+^→Fe^3+^ [38].

The relaxation Fe(2) subspectrum fitted well with Kramer’s doublet ground state in terms of a fluctuating magnetic hyperfine field at the ^57^Fe nuclei of *H*_hf_ = 520 kOe. It should be noted that the high relaxation rates Ω~10^9^ s^-1^ obtained in our experiments correspond well with any possible hyperfine fields due to the relation Ω~(*H*_hf_)^2^ [39].

## 4. Discussion

Two ferrocene derivatives FcCH_2_OH and FcCH_2_OPh were used as additives in an acid-based catalyzed polycondensation reaction to form a resorcinol–formaldehyde gel. In order to confirm the inclusion of the above-mentioned ferrocene substances in a gel–polymer structure, different spectral methods were used.

The results pertaining to the Fe content obtained by different methods varied, and those from infrared spectra were inconclusive. However, a correlation between the initial load of ferrocene units and the subsequent amount of Fe in the prepared aerogel is apparent (Table 1). It must also be taken into account that unreacted ferrocene units could be washed out during solvent exchange and CO_2_ drying because of cyclopentadienyl-related compounds, which possess a high affinity with organic solvents.

When a large amount of excess ferrocene additives was used, no gel formation was observed; only solid precipitate resulted. Only when less than 7.5 mol% of ferrocene units relative to the amount of 5-methyl resorcinol was used in a reaction did the gelation process closely resemble that produced by pure resorcinol and formaldehyde and monolith aerogels were obtained in these cases. It would appear that below a particular concentration of ferrocene units the microstructure of new polymer gels containing ferrocene, as well as the subsequent aerogels, is very similar, as in the case of pure resorcinol–formaldehyde polymer. Selected ferrocene units, which are known to be a source of electrophile particles and are especially active in acidic media, closely resemble each other and exhibit behavior similar to pure resorcinol in their formaldehyde co-condensation processes. Thus, it is plausible to expect that in these polymeric structures the ferrocene moieties would display similar alternations (irregularities) in the polymer chains. Of course, the number of such irregularities and possible units included in the structure is limited.

Porosity and surface area measurements showed (Table 2) that the resulting aerogel has a medium-range surface area and a mesoporous structure with no microporosity, which is quite common for resorcinol–formaldehyde aerogels. Disparity resulting from the use of different ferrocene units will be noticed when examining porosity: Gel 1[ferrocenyl methyl phenyl ether] has a lower surface area (both BET and Langmuir) than Gel 2 [ferrocenyl methanol]. This points to some variance in the participation of ferrocene units in the gel formation reaction and inclusion into polymer structures, as well as some divergence from common RF gels.

SEM pictures show general similarity with common RF gels, which have a grainy structure composed of linked spheres. Only minor dissimilarities in grain size are visible in the SEM pictures (Figure 1); therefore, it is reasonable to expect some variation in density, specific surface area and total volume. This may result from differences in reaction conditions of common RF gels in this study or from the active participation of added ferrocene units in the formation of sol–gel. The results can also be observed in SEM images that clearly show an increase in particle size, which also produces a lower BET surface area. A fuller exploration of this divergence from other aerogels will require more research, which has already been planned.

XDR analysis revealed smooth spectra without any sharp peaks that could indicate the absence of ordered structures in the aerogels. On that basis the Fc groups are distributed evenly in the amorphous polymeric material without aggregation and formation of metal nanoparticles.

EDS spectra exhibiting characteristic Fe peaks confirmed that iron was present at different intensities in the aerogel structure of the samples. Subsequent XPS spectral analysis and Mössbauer spectral data can only be interpreted as indicating the presence of ferrocene structures, rather than separate Fe ions in the polymer matrix.

XPS results confirmed the successful grafting of Fc units onto the material. In addition, the presence of ferrocenium could be deduced from the spectra, which is typical of these systems, either due to participation in the process of gel formation or irradiation under an X-ray beam [18]. During the polycondensation reaction the ferrocene sandwich structure remains unattached and the tail sections of the ferrocene units are involved in the reactions.

Mössbauer spectroscopy also confirmed the inclusion of ferrocene units in the aerogel structure and yielded some additional information about their characteristics. The ^57^Fe Mössbauer spectra of the 1b, 2b, 1a and 3a aerogels consist of two subspectra, corresponding to two states of iron ions. The parameters of the first subspectrum Fe(1), within the limits of experimental accuracy, coincide in both cases with the hyperfine parameters of ferrocene units Fc1 and Fc2. The Fe1 subspectra probably correspond to substituted ferrocene units in the polymers. The second subspectrum Fe(2) displays characteristic relaxation behavior that occurs when ferrocene systems undergo one-electron oxidation Fe^2+^→Fe^3+^. Regardless of the fitting model selected, the ratio of the partial contributions *I*_i_ of the Fe(2) and Fe(1) subspectra varies within a very narrow range *I*_2_/*I*_1_ = 0.66–0.87 (Table 3) for all samples. An approximate ratio of 60/40 to 55/45 for ferrocene/ferrocenium can therefore be established.

The existence of oxidation states suggests the possibility of redox behavior of the resulting aerogel. According to the IUPAC definition, this aerogel can be defined as a redox polymer carrying spatially localized ferrocene side-groups. Because the polymer backbone is non-conducting the possible applications are quite specific [40].

## 5. Conclusions

The aim of this study was to introduce ferrocene units into resorcinol–formaldehyde polymer using ordinary procedures for obtaining RF aerogels in order to produce electrochemically active material.

1. All of the aerogel samples that were obtained are relatively structurally similar despite differences in the ferrocene derivatives and their initial concentration;
2. All of the aerogel samples are amorphous substrates similar to common RF aerogels and no aggregation of substrates containing Fe in the aerogel solid phase was found in their structures.
3. All of the Fe atoms in the aerogel substrates belong to ferrocene/ferrocenium at an approximate ratio of 60/40 to 55/45;
4. The inclusion limit of ferrocene structural blocks into an aerogel was found to be ~6% wt. The distinct limitations on the amount of Fe “sandwiches” included in an aerogel polymer structure could be caused by micro-level imperfections in the initial polymer structure.

Aerogel with ferrocene/ferrocenium inclusions is likely to exhibit reversible redox sensitivity (activity) in reactions with gaseous reagents. Further studies will be undertaken in which the preparation protocol will be modified to control the inclusion of ferrocene units into the structure of the aerogel on a larger scale and to conduct experiments on reversible transformations of new aerogels based on the effects of volatile reductive and oxidizing reagents.

## Figures and Tables

**Table 1:** Content of Fe in aerogels measured by different methods (in wt%).

Theoretical /from the Load to Reaction (wt%) *	Sample No	AAS	EDS #	XPS #
1.67%	1a	1.32	1.84	1.02
4.59%	3a	1.86	2.5	1.57
1.72%	1b	1.33	2.01	0.84
3.73%	2b	2.18	4.47	1.78
4.95%	3b	1.96	5.92	3.24

* The theoretical content is calculated on the basis of input of the compound containing Fc into the reaction mixture, (1a and 3a with ferrocenyl methyl phenyl ether; 1b, 2b and 3b with ferrocenyl methanol). # Because of measurement difficulties (the material is intensively charged by static electricity) the Fe value could be underestimated (≈ 5%–10%). #XPS yields quantitative data on the content of a particular element only on the thin surface of the sample, and this must be taken into account when interpreting the results.

**Table 2:** Results of porosity analysis *.

	1a	3a	1b	2b	3b
Density (g/cm^3^)	0.12 ± 0.02	0.10 ± 0.02	0.29 ± 0.02	0.14 ± 0.02	0.12 ± 0.02
BET Surface Area-S_BET_ (m^2^/g)	124	54	240	159	70
Langmuir Surface Area-S_L_ (m^2^/g)	174	78	335	225	100
Total pore volume-V_tot_ (mm^3^/g)	344	75	128	414	366

* Micropore volume (Vmic (mm^3^/g)) was zero for every sample.

**Table 3:** Mössbauer parameters for the ^57^Fe spectra recorded at 78 K. The symbol *I* denotes the relative contribution of the particular subspectrum Fe(i); the symbol *W* stands for the width of the resonant line and Ω for the relaxation rate.

Sample	Subspectrum	δ (mm/s)	Δ (mm/s)	*W* (mm/s)	Ω × 10^9^(s^−1^)	*I* (%)
**1b**	Fe(1)	0.54(1)	2.40(1)	0.27(1)	–	55.2(9)
	Fe(2)	0.51(1)	−0.10(2)	0.36(2)	5.16(3)	44.8(8)
**3b**	Fe(1)	0.53(1)	2.38(1)	0.29(1)	–	60.1(4)
	Fe(2)	0.50(2)	−0.10(2)	0.36(1)	2.93(2)	39.9(4)
**1a**	Fe(1)	0.53(1)	2.38(1)	0.27(1)	–	53.5(1)
	Fe(2)	0.49(1)	−0.13(2)	0.37(2)	3.87(7)	46.5(2)
**3a**	Fe(1)	0.53(1)	2.38(1)	0.28(1)	–	54.1(8)
	Fe(2)	0.49(2)	−0.22	0.36(1)	4.39(5)	45.9(3)

## Supplementary Materials

The following are available online at https://www.mdpi.com/2073-4360/12/7/1582/s1, Figure S1. IR spectra of base and base-acid catalyzed 5MR-FA organic aerogels; Figure S2. IR spectra of aerogels synthesised with different Fc functional units: Sample 3a and Sample 2b; Figure S3. IR spectra of ferrocene additives used; Figure S4. TGA curves of aerogel samples; Figure S5. EDS spectrum of Sample 3a; Figure S6. X-ray diffraction curves of aerogel samples; Figure S7. XPS survey spectrum of sample 3b. Photoelectron and Auger transitions are assigned; Figure S8. 57Fe Mössbauer spectrum of monomer Fc1 and Fc2 at 298 K. Table S1. Preparation of gel with ferrocenyl methyl phenyl ether (Gel 1); Table S2. Preparation of gel with ferrocenyl methanol (Gel 2); Table S3. Peak positions (and atomic concentrations) of Fe 2p3/2, O 1s, and C 1s XPS binding energies; Table S4. Mössbauer data for monomers Fc1 and Fc2.
