# Supplementary material for: Ferrocene Introduced into 5-Methylresorcinol-Based Organic Aerogels
Source: Polymers (Basel). 2020 Jul 16;12(7):1582. doi: 10.3390/polym12071582 (PMC7407292; doi:10.3390/polym12071582)
Supplement: Supplementary file 1 [file polymers-12-01582-s001.pdf]

Supplementary material

Article

Ferrocene Introduced into 5-Methylresorcinol-Based Organic Aerogels

Ludmila V. Erkhova [1], Igor A. Presniakov [1], Michail I. Afanasov [1], Dmitry A. Lemenovskiy [1], Haojie Yu [2], Li Wang [2], Mati Danilson [3] and Mihkel Koel [4,*]

[1] Department of Chemistry, Moscow State University, Lenin Hills, 1\3, Moscow 119991, Russia;
[2] State Key Laboratory of Chemical Engineering, College of Chemical and Biological Engineering, Zhejiang University, Hangzhou 310027, China
[3] Department of Material and Environmental Technology, School of Engineering, Tallinn University of Technology, Ehitajate 5, 19086 Tallinn, Estonia
[4] Department of Chemistry and Biotechnology, School of Science; Tallinn University of Technology, Ehitajate 5; 19086 Tallinn, Estonia
* Correspondence: Mihkel.Koel@ttu.ee; Tel.: +372-6204326

Synthesis of Ferrocenyl methylphenyl ether:

0.15 g of NaOH and 8.2 mL of distilled water were placed in a one-necked flask equipped with a magnetic stirrer, the reaction flask was purged with argon and 0.3 g of phenol was added and mixed. After complete dissolution of both components, 0.9 g of *N*, *N*-dimethyl aminomethyl ferrocene iodomethylate was added. The reaction mixture was stirred under argon at 85 ℃. for 5 h. An orange oily precipitate formed during the reaction. After cooling, the reaction mixture was extracted with two portions of ether (each of 15 mL), and the precipitate was completely dissolved. The ether solution was washed twice with 10 mL of 10% sodium hydroxide solution, and then with three portions of water (15 mL each) to a neutral medium. The ether solution was dried overnight over calcined sodium sulfate and evaporated. The product is a yellow powder, weight 0.42 g, yield 59.9%, melting point 127–129 ℃.

Preparation of gels

Two types of gels were prepared: Gel 1 with ferrocenyl methyl phenyl ether (Table S1) and Gel 2 with ferrocenyl methanol (Table S2).

Table S1. Preparation of gel with ferrocenyl methyl phenyl ether (Gel 1).

Sample	Molar ratio 5-MR	Molar ratio Fc1	Mass 5-MR, mg	Mass Fc1, mg	Mass of FA solution, mg	Volume of ethanol, mL	Volume of HCl, μL

1a	0.95	0.05	135	17	281	3	235
2a	0.9	0.1	128	34	281	3	235
3a	0.85	0.15	121	50	281	3	235

Table S2. Preparation of gel with ferrocenyl methanol (Gel 2).

Sample	Molar ratio 5-MR	Molar ratio Fc2	Mass 5-MR, mg	Mass Fc2, mg	Mass of FA solution, mg	Volume of ethanol, mL	Volume of HCl, μL
1b	0.95	0.05	135	12	277	3	235
2b	0.9	0.1	128	25	272	3	235
3b	0.85	0.15	121	37	267	3	235

Optimised procedure of aerogel drying

First, the gel was introduced to liquid CO_2 at a pressure of 100 bars at 25 ℃ for 20 min, in order to fill the pores of the gel with liquid CO_2 and mix it with the solvent. The exit valve of the autoclave was then opened, allowing the liquid CO_2 to flow through the gel at a constant 100 bars at 25 ℃ for 4 h to replace the mixture of CO_2 and the solvent with CO_2. After replacement, the temperature inside the autoclave was raised to 45~50 ℃ and supercritical CO_2 extraction (SFE) was carried out for 2 h. The extraction was completed by slow depressurizing the autoclave to atmospheric pressure, and then lowering the temperature in the autoclave to ambient temperature.

IR spectra results

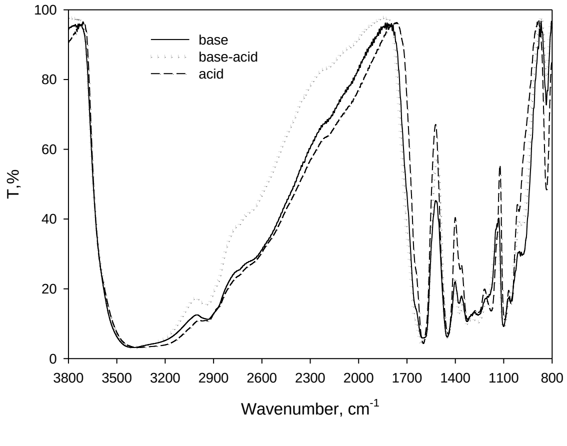

Figure S1. IR spectra of base and base-acid catalyzed 5MR-FA organic aerogels (5MR/FA = 0.5, 5MR/C = 60, W/5MR = 45) [A-L. Peikolainen, Organic Aerogels Based on 5-Methylresorcinol, PhD Thesis, TTU, 2011].

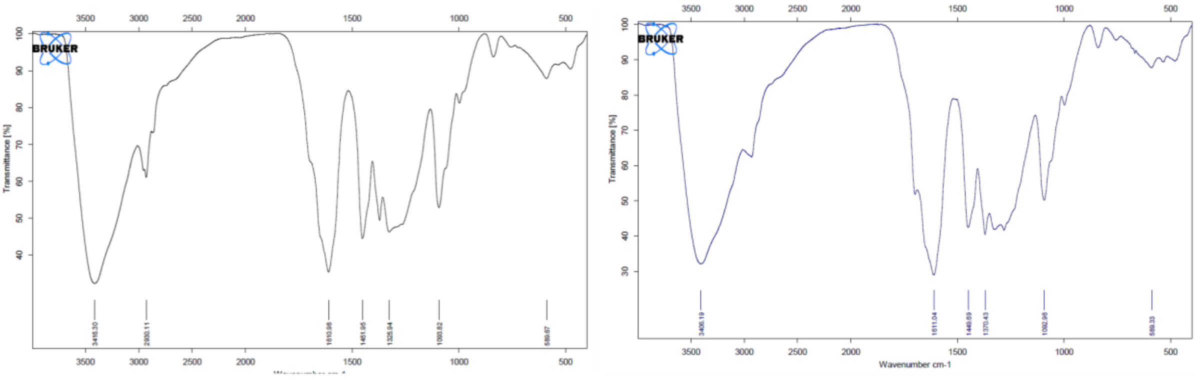

Figure S2. IR spectra of aerogels synthesised with different Fc functional units: Sample 3a and Sample 2b.

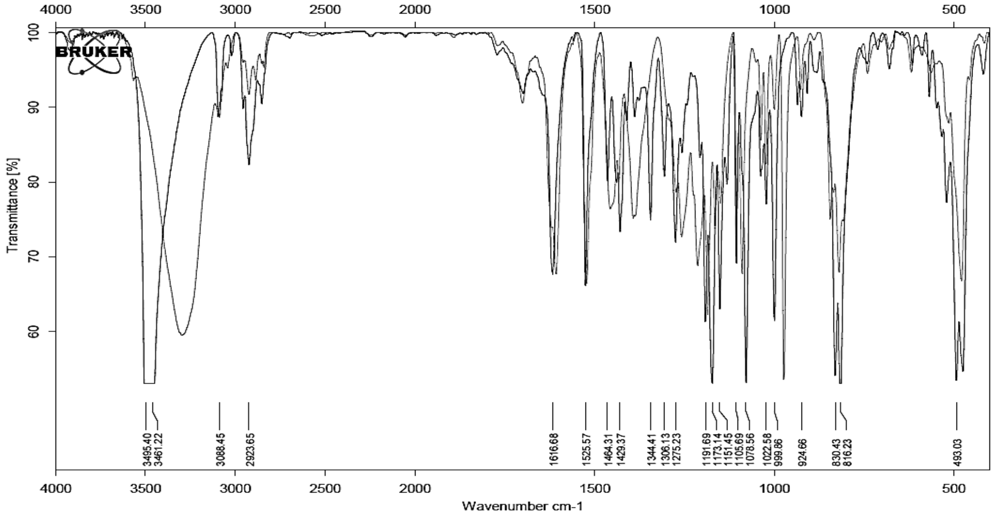

Figure S3. IR spectra of ferrocene additives used.

Comparison of Figure S2 and Figure S3 shows that main characteristic peaks of ferrocene in the range of 1000 to 2000 nm are covered with large peaks from polymer matrix.

TGA results

Instrument: Netzsh Simultaneous Thermal Analyzer (TG-DSC Apparatus).

Conditiond: N_2 atmosphere; heating rate 10 K/min, up to 900 °C

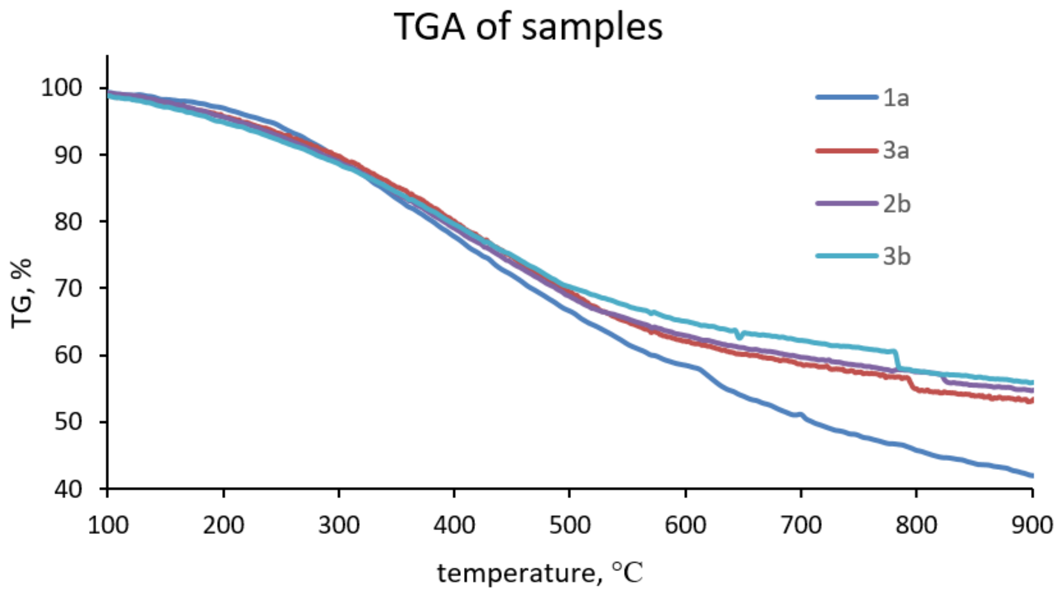

Figure S4. TGA curves of aerogel samples.

EDS analysis

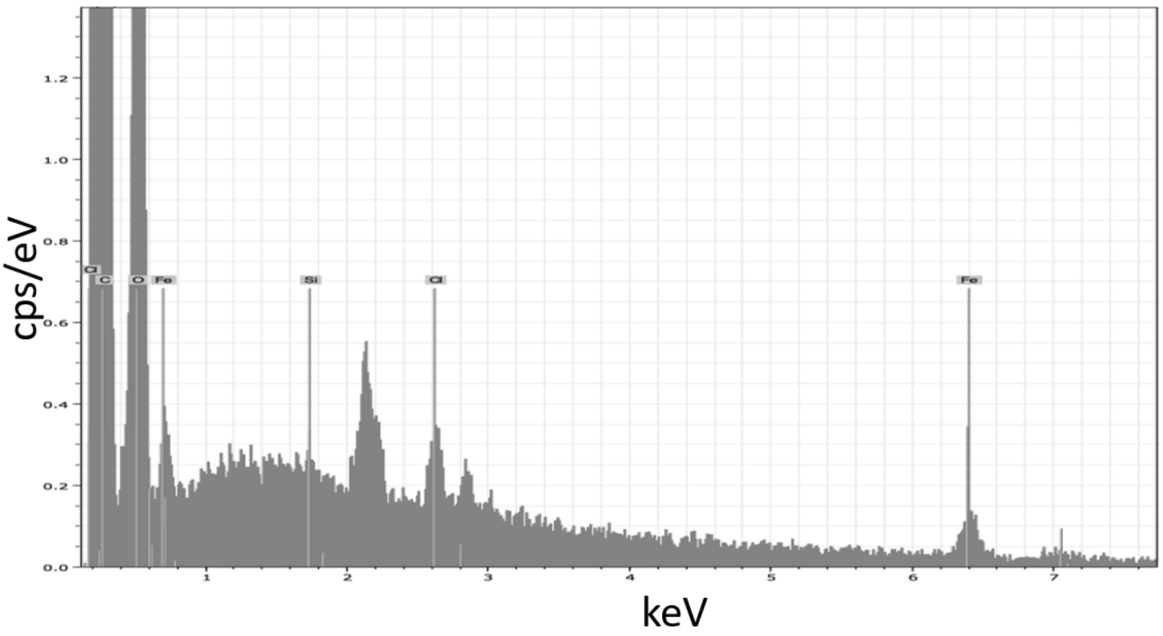

Figure S5. EDS spectrum of Sample 3a.

The X-ray diffraction (XRD) analysis

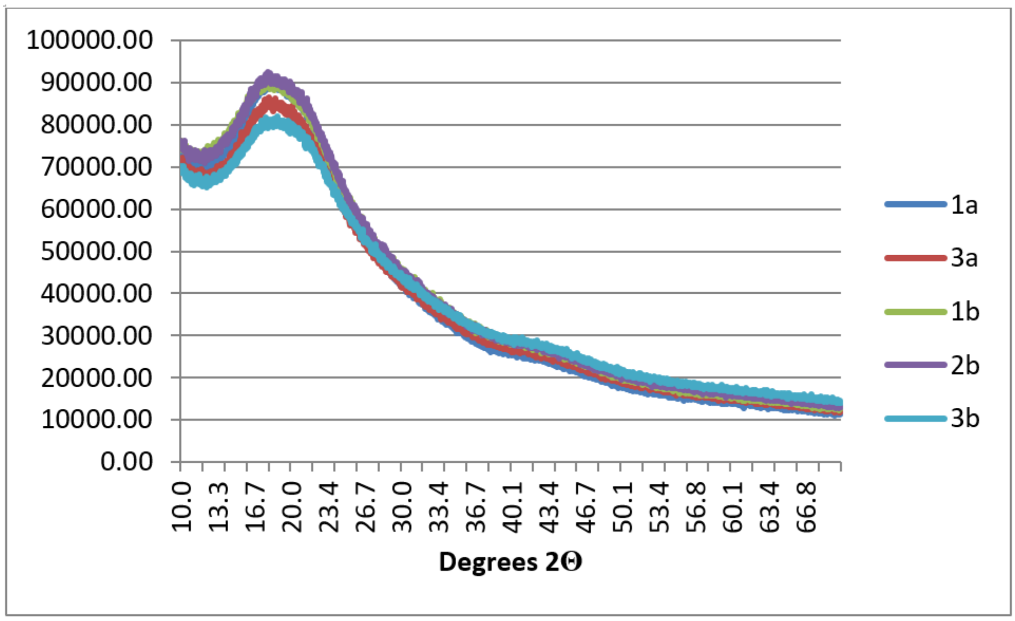

Figure S6. X-ray diffraction curves of aerogelsamples.

XPS results

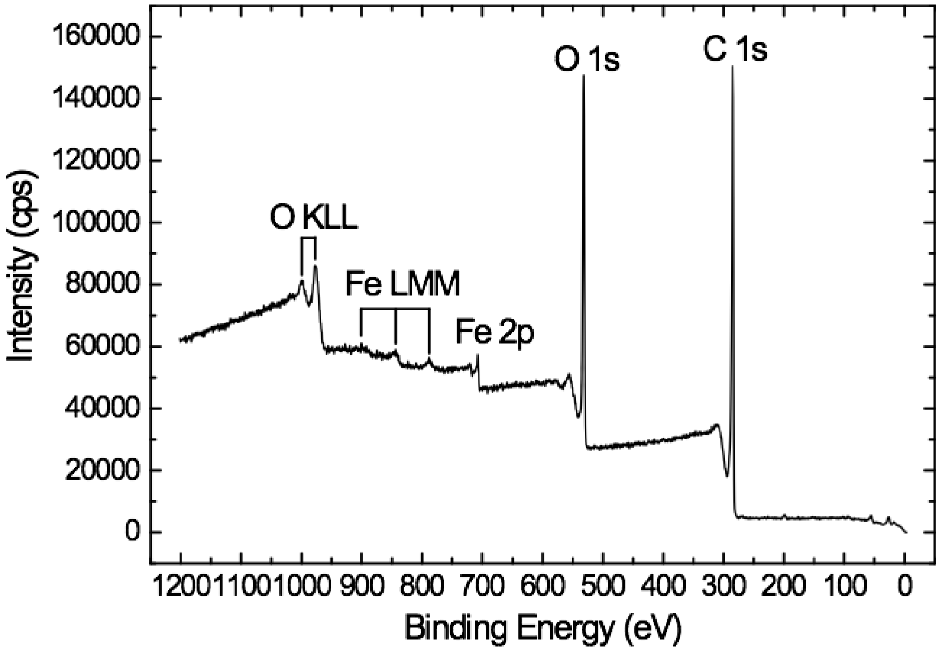

Figure S7. XPS survey spectrum of sample 3b. Photoelectron and Auger transitions are assigned.

Table S3. Peak positions (and atomic concentrations) of Fe $2p_{3/2}$, O 1s, and C 1s XPS binding energies.

	Fe $2p_{3/2}$				*O 1s*						*C 1s*									
	Fe^{2+}		Fe^{3+}		-C=O		-C-O		O-C=O		C-C		C-O		C=O		O-C=O		CO_3	
	eV	at.%	eV	at.%	eV	at.%	eV	at.%	eV	at.%	eV	at.%	eV	at.%	eV	at.%	eV	at.%	eV	at.%
3b	707.5	(0.48)	709.7	(0.29)	531.2	(2.31)	532.6	(18.40)			284.6	(51.20)	285.9	(13.83)	287.2	(9.41)	288.7	(1.97)	290.7	(2.12)
2b	707.6	(0.27)	709.7	(0.14)	531.3	(2.20)	532.7	(17.09)			284.6	(51.17)	285.9	(14.55)	287.2	(9.25)	288.5	(2.70)	290.7	(2.39)
1b	707.6	(0.11)	710.5	(0.08)	531.4	(2.01)	532.9	(18.00)			284.6	(45.55)	285.7	(20.17)	286.9	(7.24)	288.3	(4.56)	290.7	(2.29)
3a	707.3	(0.22)	709.9	(0.15)	531.4	(4.09)	532.6	(15.81)			284.6	(55.47)	286.1	(14.67)	287.5	(6.41)	288.5	(1.11)	290.7	(2.10)
1a	707.5	(0.15)	710.2	(0.09)	531.4	(2.54)	532.6	(15.53)	533.6	(1.89)	284.6	(57.29)	286.2	(13.55)	287.6	(4.24)	288.7	(2.59)	290.8	(2.15)

Mössbauer measurements

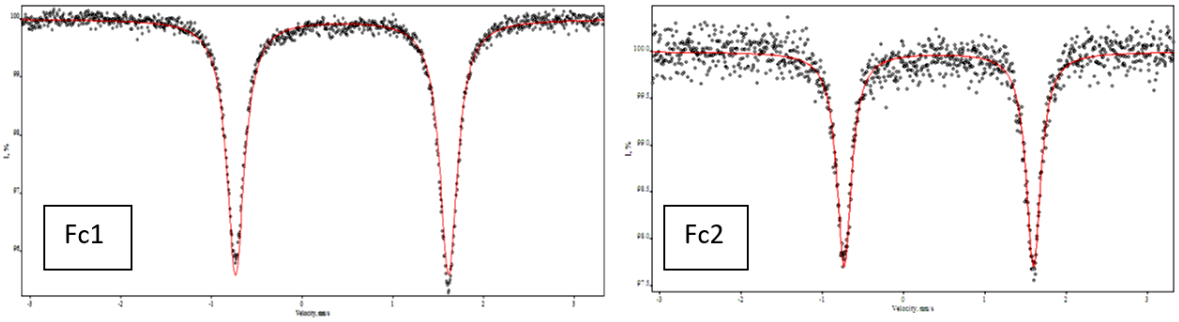

Figure S8. 57Fe Mössbauer spectrum of monomer Fc1 and Fc2 at 298 K.

Table 4S. Mössbauer data for monomers Fc1 and Fc2

monomer	T, K	δ (mm/s)	Δ (mm/s)	*W* (mm/s)
Fc1 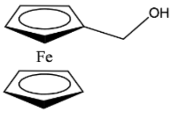	78	0.52(1)	2.38(1)	0.23(1)
	298	0.44(1)	2.35(1)	0.23(1)
Fc2 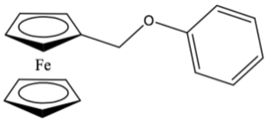	78	0.53(1)	2.38(1)	0.23(1)
	298	0.43(1)	2.34(1)	0.23(1)
